# Socioeconomic, Behavioural, and Environmental Determinants of Asthma Inequalities in Europe: A Cross-Sectional Study Using EHIS Data

**DOI:** 10.3390/ijerph23050667

**Published:** 2026-05-19

**Authors:** Anastase Tchicaya, Nathalie Lorentz, Laureen Vanni

**Affiliations:** Luxembourg Institute of Socio-Economic Research—LISER, 4366 Esch-sur-Alzette, Luxembourg; nathalie.lorentz@liser.lu (N.L.); laureen.vanni@liser.lu (L.V.)

**Keywords:** asthma, socioeconomic determinants, air pollution, environmental health, asthma prevalence, spatial heterogeneity, multilevel models, Europe, public health policy

## Abstract

**Highlights:**

**Public health relevance—How does this work relate to a public health issue?**

- Asthma is a major chronic respiratory disease and the second leading cause of death among chronic respiratory conditions worldwide.
- This study uses EHIS data to estimate the prevalence of adult asthma and examine its social, behavioural, and environmental determinants across 26 European countries.

**Public health significance—Why is this work of significance to public health?**

- Education, employment status, obesity, and air pollution significantly affect asthma prevalence in Europe.
- Socioeconomic disadvantage, high-risk behaviours, and environmental exposures interact to increase asthma risk.

**Public health implications—What are the key implications or messages for practitioners, policy makers and/or researchers in public health?**

- Variations in asthma prevalence reflect differences in individual and contextual factors across countries.
- Addressing upstream determinants is essential to reduce asthma burden and promote respiratory health equity in Europe.

**Abstract:**

Asthma is a chronic respiratory disease with uneven prevalence across population groups. This study investigated the associations between socioeconomic status, health behaviours, and environmental exposures and asthma prevalence across European countries. We conducted a cross-sectional analysis using data from the European Health Interview Survey (EHIS) covering wave III (2019). The sample included 223,453 adults aged 20 or older from 26 European countries. Asthma prevalence was self-reported. Socioeconomic variables included education and employment status, while behavioural factors included smoking and overweight status. Environmental exposures encompassed urbanisation and air pollution. Multilevel logistic regression models examined associations between asthma prevalence and its socioeconomic, behavioural, and environmental factors. Asthma prevalence was higher among individuals with lower educational attainment (OR = 1.30; 95% CI: 1.20–1.40), those who were unable to work due to long-standing health problems (OR = 2.27; 95% CI: 2.04–2.52), and retired individuals (1.44; 95% CI: 1.31–1.57). Individuals with pre-obesity and obesity had increased odds of asthma (OR = 1.13; 95%CI: 1.07–1.19, and OR = 1.76; 95%CI: 1.66–1.86, respectively). Urbanisation (OR = 1.13; 95%CI: 1.07–1.19) and exposure to air pollution (CO_2_ and PM_2.5_) were both significantly associated with higher asthma prevalence. Six countries exhibited a significant deviation from the average asthma prevalence. Inequalities in asthma prevalence in Europe were linked to socioeconomic disadvantage, unhealthy behaviours, and adverse environmental conditions. Some variability in asthma prevalence was independent of individual characteristics. These findings highlight the need for integrated public health policies that address the structural, behavioural, and environmental determinants of respiratory health.

## 1. Introduction

Asthma is a chronic respiratory disease that affects a large portion of the world’s population. It is characterised by chronic inflammation of the airways and is the second leading cause of death among chronic respiratory diseases [1,2]. Asthma has a substantial impact on quality of life [3,4,5] at all ages due to the numerous factors contributing to its occurrence and prevalence.

The global prevalence of asthma in adults was 3.3% in 2017 and projected to reach 6.6% in 2022 [6] and was estimated at approximately 4–10% in Europe in 2016 [7]. These figures mask significant variability/heterogeneity across and within different countries. To our knowledge, more recent figures on the global prevalence of asthma are not currently available.

Previous research has shown that asthma is a multifactorial disease. Low socioeconomic status, as measured by income, education, and employment, has been associated with a high risk of asthma and poorer disease control [8,9,10]. Thus, lower levels of education and income have been positively associated with respiratory disease morbidity and mortality [11,12]. Socioeconomic status (SES) may be particularly relevant for asthma because of the pathways through which it can adversely affect asthma outcomes [12].

A broader systematic review [13] found consistent evidence that indicators of socioeconomic disadvantage are associated with increased asthma risk. This systematic review included 183 observational studies covering children, adolescents, and adults from Europe, North America, Australia, and selected middle- and low-income countries (Africa and Asia). The strength of these relationships between socioeconomic position and asthma prevalence varies across countries and welfare regimes.

In some studies, education, rather than occupation, was differently associated with adult asthma phenotypes in the general population [14]. The authors hypothesise that the absence of any association with occupation could indicate that sensitisers and irritants have more complex interactions that are not easily captured by occupational classification. A more recent review, synthesising evidence from studies conducted among working-age adults across various occupational settings, reported no significant association between occupational classes and any asthma phenotype [15].

The social impact of asthma in adults represents a significant burden on society, as measured by lost workdays due to asthma in the past 12 months. According to the results of a 2004 study, the percentages of adults who lost workdays due to asthma were as follows: 25% in the United States of America (USA), 17% in Sweden, 28% in the Netherlands, 11% in Bulgaria, and 32% in the Czech Republic [16]. Occupational exposure remains another critical factor. Workers in the agriculture, manufacturing, cleaning, and healthcare fields are often exposed to irritants or allergens that can trigger or worsen asthma symptoms. While occupational asthma has historically been studied in isolation, clinical guidelines emphasise the role of workplace exposures in adults with asthma and highlight the need to consider broader contextual factors, including socioeconomic conditions, when interpreting disease patterns [17].

Behavioural factors such as smoking and overweight play an important role in the occurrence and prevalence of asthma. Previous studies have shown that smoking and exposure to smoking are associated with poor clinical outcomes in asthmatic adults [18,19,20]. Indeed, current smoking is a risk factor for the development of asthma and other adverse clinical outcomes, including increased exacerbations, accelerated decline in lung function, and high rates of morbidity [18,20]. Adults with asthma who smoke cigarettes had greater declines in lung function, worse asthma severity, less control over asthma symptoms, higher rates of hospitalisation, and a greater need for emergency medications than non-smokers with asthma [20,21].

Obesity, considered a significant risk factor for asthma in adults [3,22,23], is thought to affect factors such as poor asthma control, exacerbations, reduced lung function, increased hospitalisation rates, and mortality in people with asthma [3], and has been associated with increased asthma incidence and reduced lung function [3,23].

Although previous research has established associations between obesity and asthma, the overall understanding of this relationship remains limited, particularly in a broader context encompassing data from multiple countries and multiple asthma determinants. Many studies are limited to specific regions or populations and lack broad representativeness [24]. We therefore aimed to examine the relationship between asthma prevalence rates and weight status using population-representative data from European countries.

Environmental determinants also play a significant role in the unequal distribution of asthma in Europe. For example, urbanisation is associated with increased asthma prevalence, likely due to several lifestyle factors [19]. Urban areas with high traffic-related air pollution tend to have elevated asthma prevalence, especially among adults occupationally or residentially exposed.

According to the World Health Organisation (WHO), air pollution is a significant risk factor for asthma as it causes new cases and aggravates existing conditions. For example, different associations between average annual concentrations of PM_2.5_, SO_2_, and O_3_ and annual rates of hospitalisations and emergency department visits for asthma were found in New York State from 2005 to 2007 [25]. The association between respiratory diseases and air pollution in Europe is well-documented. Exposure to particulate matter (PM_2.5_), nitrogen dioxide (NO_2_), and ground-level ozone (O_3_) has been associated with increased asthma symptoms, hospitalisations, and mortality in both children and adults, particularly in urban areas of Europe, North America, and Asia, based on studies conducted over the past two decades [26].

Climate change, mediated by greenhouse gases (CO_2_), causes adverse health effects in the most vulnerable patient populations, such as the elderly, children, patients with chronic non-communicable diseases, and those in distressed socioeconomic strata [27,28].

The present study aimed to examine the influence of different factors—namely, SES (level of education and employment status), behaviour (smoking and weight status), and the environment (degree of urbanisation and air pollution)—on the prevalence of asthma across European countries participating in wave III (2019) of the European Health Interview Survey (EHIS). We also assess the magnitude of heterogeneity in asthma prevalence, independent of individual characteristics, across a large population.

## 2. Materials and Methods

### 2.1. Design

We designed this cross-sectional study to provide an evidence-based overview of asthma prevalence and its determinants in Europe using data from the EHIS, covering all European countries that participated in wave III of the survey and providing a comprehensive multi-country perspective (see Table A1). To understand the influence of environmental risks by country of residence on asthma prevalence, we added information on air pollution levels in each country to the database (see Table A2).

### 2.2. Data and Data Sources

The data were obtained from wave III of the EHIS, conducted in 2019, across all EU countries, which consists of four modules: health status, healthcare use, health determinants, and socioeconomic background variables. The EHIS was a cross-sectional survey targeting people aged 15 or older living in private households, meaning that people residing in institutions such as hospitals, retirement homes, nursing homes, or long-term care facilities were not eligible for the interview. This study focused on adults, so analyses were restricted to individuals aged 20 years and older. Age information was provided only in grouped categories, which varied across countries; for example, in Iceland and Malta, the first reported age group was 15–19 years.

Furthermore, this study used aggregated data at the country level from other sources on climate and environmental risks (carbon dioxide emissions, particulate matter, nitrogen dioxide, and ozone).

### 2.3. Main Outcomes

Asthma prevalence was considered the primary outcome of this study. It was measured by determining whether patients had asthma in the past 12 months (including allergic asthma), defined as yes or no.

### 2.4. Sociodemographic Variables

The sociodemographic variables included age, gender, and marital status. Age was available only as a categorical variable, defined according to predefined age groups. Gender was classified as male or female, and marital status was categorised as single, married or cohabiting, divorced/separated, or widowed.

Educational level and employment status were used as indicators of socioeconomic status. Educational level was defined as the highest level of education, based on the International Standard Classification of Education (ISCED) adopted by the United Nations Educational, Scientific and Cultural Organization (UNESCO) [29], and categorised into primary (ISCED 0 and 1), secondary (ISCED 2, 3, and 4), or tertiary education (ISCED 5, 6, 7, and 8). Employment status was defined as employee, unemployed, retired/disabled, fulfilling domestic tasks, unable to work due to long-standing health problems, or other.

### 2.5. Behavioural Health Risk Factors

Health risk factors included smoking and having overweight or obesity. These health risk factors were selected for their likely influence on asthma occurrence.

Smoking behaviour was defined as daily smoking, occasional smoking, or no smoking.

Obesity status was based on self-assessed weight and height measurements, which people used to calculate their body mass index (BMI). People with a BMI < 25 kg/m^2^ were classified as having normal weight; those with a BMI ≥ 25 kg/m^2^ and <30 kg/m^2^ were considered as having overweight; and those with a BMI > 30 kg/m^2^ were considered as having obesity.

### 2.6. Location of Residence

Location of residence defined the neighbourhoods in which people lived and was categorised by degree of urbanisation as follows: cities, towns, suburbs, and rural areas.

### 2.7. Climate and Environmental Risks

Carbon dioxide (CO_2)_, fine particulate matter (PM_2.5_), and nitrogen dioxide (NO_2_) are three pollutants that are often present in the air at the same time. Several recent studies have associated air pollution with the asthma prevalence rate [30,31,32,33,34].

The carbon dioxide emission index is an indicator of the Sustainable Development Goal (SDG) 9.4 in the United Nations SDG framework and is a measure of carbon intensity. It reports the quantity of carbon dioxide emitted per unit of economic value (kilograms of CO_2_ emitted per dollar of Gross Domestic Product) [35]. CO_2_ is a greenhouse gas primarily linked to emissions from the combustion of fossil fuels contributing to global warming, and international targets have been set to reduce CO_2_ emissions to limit climate change.

The WHO guidelines state that the limit of fine particulate matter (PM_2.5_) is 10 µg/m^3^ (micrograms per cubic metre), but the EU guidelines set an annual mean air quality limit of 25 µg/m^3^ for PM_2.5_ in 2015, which is 2.5 times higher [36]. PM_2.5_ is defined as small airborne particles, emitted mainly by vehicles, power plants, factories, and industrial activities.

Nitrogen dioxide is an air pollutant emitted mainly by motor vehicles and industrial installations (the average annual amount is NO_2_ ≤ 40 µg/m^3^, according to EU air quality standards). According to the WHO guidelines, the annual average NO_2_ concentration is 10 µg/m^3^. Studies have suggested that exposure to NO_2_ may contribute to asthma by causing chronic inflammation [30,31,37].

Long-term exposure to ozone (O_3_), a seasonal pollutant, was associated with an increased risk of developing asthma in adults and a decline in respiratory function [31,38,39].

### 2.8. Statistical Analysis

A descriptive analysis was conducted to measure asthma prevalence by country, gender, and education level. The characteristics of the dataset, including asthma prevalence, socioeconomic, and environmental factors, and health risk factors, were described.

We used measures of inequality, such as differences in asthma prevalence and prevalence ratios, to estimate the magnitude of socioeconomic inequalities in asthma for each gender. Differences in prevalence are observed in the asthma rates of people with primary education compared with those with tertiary education.

We performed multilevel logistic regression models while considering both the fixed effects of socioeconomic status, health risks, and location of residence on asthma prevalence in adults and the specific characteristics of countries in which people live, especially air pollution (carbon dioxide emission index, PM_2.5_, NO_2_, and O_3_), which allowed us to measure random effects. These statistical methods also allowed us to analyse the spatial heterogeneity in asthma prevalence and its determinants. All models were estimated using data weighted according to the weights provided in the EHIS database. Given the cross-sectional design, the analyses focused on association rather than causal relationships.

Analyses were conducted for all countries included in wave III of the survey. The results were assessed at a significant threshold of error of 5% or a 95% confidence interval (95% CI).

Intraclass correlation coefficients (ICCs) were computed to assess the homogeneity in asthma prevalence within individuals and countries from the empty model. This allowed us to assess the proportion of variation in asthma prevalence based on country of residence rather than individual characteristics [40,41].

Thus,

$$ICC=\frac{Variance\;between\;countries(u_{0j})}{Variance\;between\;countries\left(u_{0j}\right)+Variance\;intra-individual(\frac{\pi^{2}}{3})}$$

where $\;\frac{\pi^{2}}{3}$ represents the within-individual variance in a logistic regression model.

Theoretically, the ICC ranges from 0 to 1, but it is typically less than 0.2 (and more commonly around 0.01–0.05) in randomised cluster trials [42].

All statistical analyses were performed using the SAS 9.4 software (SAS Institute Inc., Cary, NC, USA), and multilevel logistic regression models were realised using the SAS Procedure GLIMMIX.

## 3. Results

Analyses were conducted on a sample of 26 European countries comprising 223,453 individuals aged 20 and over (51.9% of whom were women) using data from Wave III (2019) of the EHIS. These analyses allowed us to measure the extent of asthma prevalence according to individual characteristics across all countries and in relation to inequalities linked to education level within each country; second, it allowed us to quantify the influence of various socioeconomic, behavioural, and environmental determinants of inequality in asthma prevalence by measuring fixed and random effects.

### 3.1. Asthma Prevalence According to Individual and Environmental Characteristics for All Countries Considered

The prevalence of asthma was higher among women, in individuals with a primary education, widowers, in individuals unable to work due to long-term health problems, non-smokers, and individuals with obesity (see Table 1).

The prevalence of asthma was 5.5% in women versus 4.0% in men, 6.5% in those aged 70 and over versus 4.3% in young people aged 20–29, 6.8% in those with a primary education versus 4.3% in those with a tertiary education, 4.8% in unemployed individuals versus 3.9% in employed individuals, and 7.1% in individuals with obesity versus 4.0% in those with normal weight.

Regarding environmental factors, the prevalence rates of asthma were 5.6% (CO_2_), 6.6% (PM_2.5_), 6.8% (NO_2_), and 6.4% (O_3_) among people living in countries with moderately low air pollution levels compared to 4.4%, 3.6%, 4.5%, and 4.5%, respectively, among those living in countries with higher pollution levels. 223453.

### 3.2. The Spatial Distribution of Asthma Prevalence

Table 2 shows the prevalence of asthma and the distribution of asthma prevalence by education level in each participating country.

The overall asthma prevalence ranged from 1.6% (Romania) to 10.3% (Iceland). Specifically, it ranged from 6% to 10.3% in seven countries (Malta, the Netherlands, Ireland, Denmark, Sweden, Norway, and Iceland) and was less than 6% in all other countries. The distribution of asthma prevalence by education level was uneven across most of the countries considered, to the detriment of those who had only completed primary school. This unequal distribution was statistically significant in Austria, Belgium, Bulgaria, Cyprus, the Czech Republic, Denmark, Estonia, Greece, Spain, Croatia, Hungary, the Netherlands, Poland, Portugal, Romania, Serbia, Slovenia, and Slovakia. Among these countries, the disparity in asthma prevalence between individuals with primary and tertiary education was highest in Romania (6.20%), Slovakia (5.36%), Serbia (3.59%), the Czech Republic (3.26%), Hungary (3.23%), and Austria (3.00%).

In countries such as Malta, Iceland, Lithuania, Luxembourg, Sweden, Norway, Latvia, and Ireland, the distribution of asthma prevalence according to education level was not statistically significant.

When analysed by gender, only five countries (Greece, the Netherlands, Poland, Romania, and Serbia) showed a social gradient in asthma prevalence among both men and women (see Table A3).

The asthma prevalence ratio between individuals with primary and higher education reveals significant social disparities in the disease across several European countries, including Romania, Serbia, Slovakia, Hungary, Austria, Belgium, Greece, the Netherlands, and Bulgaria. This ratio highlights social inequality in risk factors for asthma exposure.

### 3.3. Socioeconomic and Contextual Determinants in Inequalities of Asthma Prevalence

Table 3 presents estimates of the influence of socioeconomic and environmental factors on asthma prevalence in Europe.

The empty model (i.e., without explanatory variables) had a constant (intercept) value of 0.05, which was statistically significant. This corresponds to a predicted probability of having asthma in a typical country of 51.25%. The variance was 0.1671, corresponding to a 4.83% variability in the average asthma prevalence across countries.

Model 1 estimated the magnitude of associations between asthma prevalence and socioeconomic factors, adjusted for sex, age, and marital status. Educational attainment and employment status were significantly associated with asthma prevalence. Individuals with primary education were more likely to have asthma than those with higher education (OR = 1.37; 95% CI: 1.27–1.47). The risk of having asthma was highest among individuals unable to work due to health problems (OR = 2.41; 95% CI: 2.19–2.65), retirees (OR = 1.52; 95% CI: 1.39–1.64), and unemployed individuals (OR = 1.33; 95% CI: 1.22–1.46).

Model 2 complemented Model 1 by adding two selected behavioural factors (smoking and obesity) and the degree of urbanisation in places of residence. Smoking status was not associated with the prevalence of asthma, while obesity had a significant association. Individuals with obesity were more likely to have asthma (OR = 1.76; 95% CI: 1.66–1.86) compared to those of normal weight. The association between the degree of urbanisation and asthma prevalence was statistically significant (OR = 1.13; 95% CI: 1.07–1.20).

In Model 3, considered the complete model, environmental factors related to air pollution (CO_2_, PM_2.5_, NO_2_, and O_3_) were added. Individuals with primary and secondary education were more likely to have asthma than those with higher education (OR = 1.30, 95% CI: 1.20–1.40, and OR = 1.08, 95% CI: 1.03–1.14, respectively).

Individuals unable to work due to long-term health problems (OR = 2.27, 95% CI: 2.04–2.52), retirees (OR = 1.44, 95% CI: 1.31–1.57), and unemployed individuals (OR = 1.33, 95% CI: 1.20–1.46) were more likely to have asthma than employed individuals.

Individuals with pre-obesity (OR = 1.13, 95% CI: 1.07–1.19) and obesity (OR = 1.76, 95% CI: 1.66–1.86) were more likely to have asthma than normal-weight individuals.

The degree of urbanisation of places of residence was a risk factor for developing asthma. Individuals living in densely or moderately populated areas were more likely to have asthma (OR = 1.13, 95% CI: 1.07–1.19) than those living in rural or very sparsely populated areas.

Individuals living in countries with higher carbon dioxide levels (>0.925) were less likely to have asthma (OR = 0.66, 95% CI: 0.49–0.88) than those living in countries with lower carbon dioxide levels (≥0.900). Air pollution was statistically significantly associated with asthma prevalence for carbon dioxide and fine particulate matter (PM_2.5_) (OR = 0.54, 95% CI: 0.38–0.78).

Moreover, results were similar when estimates were computed without weights (see Table A4).

### 3.4. Spatial Heterogeneity in Asthma Prevalence Highlighted by Random Effects

The random effects measure indicates the magnitude to which a specific country’s average asthma prevalence deviates from the global average, as measured by multilevel modelling.

The results presented in Table 4 reveal spatial heterogeneity in asthma prevalence across six countries (Denmark, Estonia, Latvia, Malta, Romania, and Sweden), each exhibiting a statistically significant deviation from the average.

Note that the random effects are centred around the corresponding fixed effects around the intercept. Hence, countries with a positive random effect (such as Denmark, Malta, and Sweden) have a greater average asthma prevalence than that of the countries overall (=the intercept), and countries with a negative random effect (such as Estonia, Latvia, and Romania) have a lower prevalence compared to the average across all countries.

## 4. Discussion

This cross-sectional study examined the socioeconomic, behavioural, and environmental determinants of asthma prevalence across multiple European countries, using individual data from wave III of the EHIS and aggregated environmental data. The findings suggest significant inequalities in asthma distribution, with a consistently higher prevalence observed among individuals with lower socioeconomic status, those with high-risk factors such as obesity, and those exposed to adverse environmental conditions.

The descriptive analysis results show that the prevalence of asthma was higher among women, older people, retirees, people unable to work, non-smokers, and individuals with obesity, consistent with other studies [43,44,45].

Similarly, asthma prevalence was higher among people living in countries with lower concentrations of air pollutants (CO_2_, PM_2.5_, NO_2_, and O_3_). This a priori result was surprising, but it could be the consequence of a more complex dynamic due to the multifactorial nature of asthma. Some studies have shown that asthma prevalence in adults is often higher in high-income countries, which tend to have better air quality and therefore lower pollutant concentrations [46]. In fact, such differences could be linked to asthma diagnosis rates, asthma awareness, access to care, and environmental, behavioural, and genetic factors.

Regarding the geographical distribution of asthma prevalence by country and education level, significant disparities were observed both between and within European countries depending on individuals’ socioeconomic status. Prevalence rates were 1.6% in Romania, 4.1% in Spain, 6.4% in the Netherlands, and 10.3% in Iceland. Social inequalities in asthma prevalence were more pronounced within each country, with a negative social gradient observed in most European countries. For example, individuals with primary education had asthma prevalence rates 6.2 times higher than those with higher education in Romania, 5.4 times higher in Slovakia, 3.6 times higher in Serbia, 3.2 times higher in Hungary, 3.0 times higher in Austria, and 2.2 times higher in Poland.

These descriptive analysis results were further developed using multilevel statistical models, given the multifactorial nature of asthma determinants.

Our analysis confirms that low socioeconomic status—with education and employment used as a proxy for SES—is strongly associated with increased asthma prevalence. These results align with previous European studies showing that social disadvantage increases vulnerability to asthma through multiple pathways, including reduced healthcare access, higher psychosocial stress, and increased exposure to environmental hazards [13,47].

Our results show a strong inverse social relationship between education level and asthma prevalence, both overall and at a smaller scale within each European country. Indeed, the higher the education level, the lower the asthma prevalence. These results are consistent with previous studies that found statistically significant associations between low socioeconomic status and asthma prevalence, including poor disease control [12,14,48,49]. In fact, a low education level was associated with a high risk of asthma incidence and prevalence, as well as respiratory symptoms [48,49].

Regarding the links between employment status and asthma prevalence, our results show significant differences across occupational types. The asthma prevalence rate was 2.3 times higher among adults unable to work due to long-term health problems, 1.3 times higher among unemployed individuals, and 1.4 times higher among retirees compared to employed individuals.

However, this association between employment status and asthma was less evident in the literature. In a recent study [48], none of the employment categories were associated with asthma phenotypes in adults. This, according to the authors, could stem from the fact that none of the asthma phenotypes were primarily linked to employment or occupation.

The associations observed in our study between employment status and asthma prevalence should not be interpreted as causal. The available data did not allow us to determine whether the asthma conditions of the individuals studied were work-related or exacerbated by work. Previous studies have also reported inconsistent associations between occupational classification and asthma [50,51].

Numerous observational studies have shown that smoking is associated with asthma and poor clinical outcomes, including suboptimal asthma control, more exacerbations, and greater use of asthma-related healthcare services [18,20,21]. These findings support the hypothesis that lifestyle behaviours not only contribute to the onset of asthma but may also worsen existing symptoms, particularly when compounded by other stressors. However, our results did not show a statistically significant association between smoking status and asthma prevalence as in some studies and reviews [52,53,54,55]. In a population-based study of Californian adults [52] there was also no association between asthma and the risk of current smoking after controlling for covariates (OR 1.1; 95%CI 0.8, 1.6).

We also found that weight status was significantly associated with asthma prevalence. This positive relationship translates into a high prevalence of asthma in pre-obese and obese adults compared to adults of normal weight (OR: 1.13 and OR: 1.76, respectively). These results are consistent with those of other studies [49,56,57,58,59]. For example, in a meta-analysis of seven prospective studies involving over 300,000 adults, examining the dose–response relationship between obesity and asthma, the authors found odds ratios for asthma onset of 1.38 in overweight groups and 1.92 in obesity groups compared to normal-weight groups [57].

The results support the conclusion that increased BMI is associated with a higher prevalence of asthma [60].

In a 2019 study using Korean data on men and women aged 40 and over, the risks of developing asthma were 1.40 and 1.23, respectively, in men and women with obesity compared with those with a body weight between 18.5 kg/m^2^ and 23 kg/m^2^ [61].

Our results are therefore largely consistent with those found in several studies outside of the European context.

The environmental dimension further amplified disparities in asthma prevalence in European countries.

In this study, adults living in cities or densely populated areas were more likely to report having asthma than those living in rural or sparsely populated areas, which is consistent with the results of previous studies [62]. Urbanisation was associated with the prevalence of asthma and other respiratory diseases, primarily due to the high levels of air pollutant emissions characterising their development [62]. Asthma prevalence was higher in urban than in rural areas, and the rate of urbanisation was significantly associated with asthma [63]. However, a study conducted in Spain [64] found no significant difference in asthma prevalence between urban and rural areas.

In the absence of air pollution data directly linked to place of residence, we supplemented the analysis of the influence of place of residence on asthma prevalence by adding data on air pollutant concentration levels in the country of residence. Individuals living in countries with high concentrations of various pollutants would likely have a greater risk of suffering from asthma than those living in countries with low pollutant concentrations. Since the carbon dioxide emissions index is one of the indicators used by the United Nations to measure climate change, it was necessary to assess its influence on respiratory diseases by examining asthma prevalence in our sample of European countries. Our results show a statistically significant relationship between carbon dioxide emissions and asthma prevalence.

Nevertheless, this association was the opposite of what might have been expected. Specifically, individuals living in countries with low carbon dioxide emissions were more likely to suffer from asthma than those living in countries with high emissions. The inverse relationship between air pollution and asthma prevalence observed in this study may represent a paradox rather than a true protective effect. A recent systematic review and meta-analysis by Lee et al. [31] reported substantial heterogeneity across studies. For example, only 39% of studies (7/18) examining PM_2.5_ exposure and incident adult asthma found a significant positive association, while 32% (6/19) reported similar findings for other exposure metrics.

By contrast, climate change mediated by greenhouse gases has been shown to cause adverse health effects, including increased prevalence of allergic respiratory diseases, exacerbations of chronic obstructive pulmonary disease, premature mortality, and declines in lung function among the most vulnerable populations [62].

The relationship between asthma and fine particulate matter (PM_2.5_) has been explained by high levels of road traffic, sports activities near road traffic, and vehicle emissions [33,63,65]. The results of our study, excluding these parameters from our models, indicate consistency in the association between country-level exposure to fine particulate matter and asthma prevalence. However, there was a greater likelihood of asthma in countries with the lowest fine particulate matter (PM_2.5_) concentrations (≤10) than in those with average levels (≤10, 25). The multilevel regression analysis results confirm those obtained with the descriptive analysis. Unlike the results of large international studies [46], which included several countries from different continents and therefore different levels of development and income, our results were obtained solely from a sample of European countries. Despite this, the risk of a higher prevalence of asthma in countries with better air quality compared to others remained significant for both carbon dioxide and fine particulate matter. The explanation is complex, as differences in diagnostic capacity, reporting, and access to healthcare are often cited [46,66].

Furthermore, we did not find statistically significant associations between pollutants (such as nitrogen dioxide and ozone) and asthma prevalence. This result is consistent with those reported in a recent literature review on the relationship between ozone and asthma incidence [31]. The authors of this review considered the evidence to be inconsistent.

Overall, our findings reveal marked heterogeneity in asthma prevalence across the countries included in the analysis. This variability likely reflects differences in environmental exposures, healthcare systems, and socioeconomic or behavioural determinants that influence asthma outcomes. Nevertheless, only a few countries (Estonia, Denmark, Latvia, Malta, Romania, and Sweden) showed statistically significant deviations from the overall average prevalence, suggesting that, while contextual heterogeneity exists, the magnitude of between-country disparities may be modest. Countries exhibiting positive random effects relative to the overall mean prevalence (Denmark, Malta, and Sweden) merit particular attention as they may indicate underlying contextual or environmental factors associated with higher asthma risk.

### Strengths and Limitations

The strengths of this study include a large, diverse, population-based sample; the integration of multilevel determinants; and the use of robust statistical models adjusting for covariates. The use of harmonised data from multiple European countries strengthens our findings and supports the generalizability of these associations across different healthcare systems and policy contexts. Additionally, the inclusion of socioeconomic, behavioural, and environmental variables allowed for a comprehensive analysis of the multifactorial contributors to asthma.

Indeed, using multilevel regression methods, this study allowed us to measure the variability in asthma prevalence across countries, distinguishing between variability attributable to individual characteristics and that associated with specific country contexts.

Our findings are broadly consistent with previous studies and reviews, regardless of whether weighted or unweighted data were used.

However, several limitations must be acknowledged. Self-reported asthma diagnosis and exposures may introduce reporting bias [67]. In addition, some variables, such as pollutant levels or allergen exposures, were not measured at the individual level but were only available at the national level, limiting the assessment of their contribution. An inverse association between air pollution and asthma prevalence has been reported in systematic reviews and meta-analyses, whereby higher prevalence is observed in lower-pollution countries. This pattern may reflect spatial misalignment between exposure and outcome measurement, rather than a protective effect of pollution. In this context, differences in reporting and diagnosis in the EHIS may further contribute to misclassification of asthma prevalence but are unlikely to fully explain the observed cross-country pattern. Accordingly, the associations observed in this study cannot be interpreted as causal. These findings may also reflect an ecological fallacy, whereby inferences about individual-level relationships are drawn from aggregated exposure data [68]. Similar limitations have been noted in previous studies [12,69]. Nevertheless, certain limitations related to the specific characteristics of different countries, such as differences in access to healthcare, diagnostic practices, and environmental monitoring, could be mitigated by using multilevel regression models.

## 5. Conclusions

This cross-sectional study, based on data from 26 European countries participating in the third wave of the EHIS, highlighted the significant associations between socioeconomic disadvantage, unhealthy behaviours, adverse environmental exposures, and asthma prevalence across Europe. Our findings suggest a social gradient in asthma, with individuals who are less educated, unemployed, and living with obesity experiencing a higher burden.

The range of social, behavioural, and environmental determinants considered in this study highlights the multidimensional nature of asthma. These findings suggest that addressing asthma inequalities may require approaches that extend beyond individual-level interventions. This includes broader public health and social policies aimed at improving air quality notably in urban areas, promoting healthy lifestyles, and reducing social inequalities across Europe.

Future research should leverage longitudinal data to better assess potential causal relationships between specific factors and asthma. Such data could help inform the development of more effective prevention and management strategies. Addressing the structural determinants of health disparities remains important for reducing the burden of asthma and improving respiratory health equity across European populations.

## Figures and Tables

**Table 1:** Individual and environmental characteristics of asthma prevalence in Europe.

	n	%	Prevalence of Asthma
Sex			
Male	107,465	48.1	4.0
Female	115,988	51.9	5.5
*p*-value			<0.0001
Age-group			
20–29	32,084	14.4	4.3
30–39	38,710	17.3	4.0
40–49	40,985	18.3	4.1
50–59	38,641	17.3	4.5
60–69	35,134	15.7	5.2
70+	37,899	17.0	6.5
*p*-value			<0.0001
Marital status			
Never married	59,649	26.8	4.7
Married	125,654	56.4	4.3
Widowed	20,153	9.1	6.6
Divorced	17,321	7.8	5.7
*p*-value			<0.0001
Education			
Primary	25,861	11.7	6.8
Secondary	127,166	57.3	4.6
Tertiary	68,757	31.0	4.3
*p*-value			<0.0001
Labour status			
Employed	122,383	55.1	3.9
Unemployed	12,956	5.8	4.8
Retired	56,715	25.6	6.1
Unable to work due to long-standing health problems	5517	2.5	10.5
Fulfilling domestic tasks	10,484	4.7	4.2
Other	13,926	6.3	5.0
*p*-value			<0.0001
Type of smoking behaviour			
Daily smoking	41,868	19.3	4.0
Occasional smoking	10,388	4.8	4.6
No smoking	164,859	75.9	5.0
*p*-value			<0.0001
Obesity status			
Normal weight	91,239	43.7	4.0
Pre-obese	80,162	38.4	4.3
Obese	37,435	17.9	7.1
*p*-value			<0.0001
Degree of urbanisation			
Densely populated area	84,530	40.1	4.9
Moderately populated area	63,182	29.9	5.1
Thinly populated area	63,299	30.0	4.4
*p*-value			<0.0001
Carbon dioxide (CO_2_)			
≤0.900	63,040	28.2	5.6
]0.900–0.925]	49,193	22.0	4.5
>0.925	111,220	49.8	4.4
*p*-value			<0.0001
Particulate Matter (PM_2.5_)			
≤10	61,900	27.7	6.6
]10–25]	141,282	63.2	4.1
>25	20,271	9.1	3.6
*p*-value			<0.0001
Azote dioxide (NO_2_)			
≤10	26,915	12.1	6.8
]10–20]	138,290	61.9	4.5
>20	58,248	26.1	4.5
*p*-value			<0.0001
Ozone (O_3_)			
≤2500	55,784	25.0	6.4
]2500–5000]	102,229	45.8	4.0
>5000	65,440	29.3	4.5
*p*-value			<0.0001

**Table 2:** Geographic distribution of asthma prevalence by country according to education level and social inequalities for each country.

	All	Primary	Secondary	Tertiary	*p*-Value	Difference P-T	Ratio P/T
Austria	4.3	10.7	4.6	3.6	<0.0001	7.12	3.00
Belgium	5.6	7.8	5.9	4.6	0.0006	3.00	1.70
Bulgaria	2.3	5.4	2.1	2.1	<0.0001	3.00	2.59
Cyprus	4.1	6.8	4.0	3.2	<0.0001	3.40	2.14
Czech Republic	4.7	9.7	5.2	3.0	0.0007	2.40	3.26
Denmark	7.3	7.2	8.8	6.0	0.0003	0.80	1.22
Estonia	4.0	6.2	4.5	3.1	0.0473	3.20	2.00
Greece	3.4	5.6	3.2	2.2	<0.0001	3.30	2.59
Spain	4.1	4.8	3.9	3.7	0.0062	1.20	1.29
Croatia	4.8	8.1	4.5	4.4	0.0044	3.60	1.82
Hungary	5.1	14.1	5.0	4.4	<0.0001	9.20	3.23
Ireland	7.2	8.5	7.2	7.0	0.3061	2.10	1.23
Iceland	10.3	11.6	9.6	9.8	0.2324	1.40	1.19
Lithuania	2.8	5.4	2.7	2.7	0.1494	1.50	1.99
Luxembourg	5.9	8.1	5.3	6.1	0.2273	1.10	1.33
Latvia	3.8	5.8	4.1	3.0	0.059	2.10	1.96
Malta	6.0	5.7	6.8	6.1	0.5629	−0.40	0.94
Netherlands	6.4	10.5	6.7	4.8	<0.0001	4.60	2.17
Norway	7.8	4.0	8.4	7.2	0.1712	−3.20	0.55
Poland	4.1	7.4	3.9	3.3	<0.0001	3.90	2.25
Portugal	5.9	7.5	4.6	5.1	<0.0001	2.40	1.46
Romania	1.6	4.3	1.5	0.7	<0.0001	3.30	6.20
Serbia	3.7	8.5	3.5	2.4	<0.0001	6.00	3.59
Sweden	7.4	8.4	7.7	7.0	0.3567	1.40	1.20
Slovenia	4.8	8.7	5.6	4.2	0.0031	4.40	2.05
Slovakia	4.2	18.1	4.3	3.4	0.0013	7.40	5.36

**Table 3:** Estimates of socioeconomic and environmental determinants of asthma prevalence in European countries (wave 3, 2019), based on weighted data.

Variables	Model 0		Model 1		Model 2		Model 3	
	OR	95%CI	OR	95%CI	OR	95%CI	OR	95%CI
Fixed Effects								
Intercept	0.05 ***	0.04–0.06	0.03 ***	0.02–0.04	0.02 ***	0.02–0.03	0.04 ***	0.03–0.06
Sex (ref = Male)								
Female			1.36 ***	1.30–1.41	1.43 ***	1.36–1.49	1.43 ***	1.36–1.49
Age-group (ref = 30–39)								
20–29			1.01	0.93–1.09	1.05	0.96–1.14	1.05	0.96–1.14
40–49			1.04	0.96–1.11	1.20	0.94–1.10	1.02	0.94–1.10
50–59			1.04	0.97–1.12	1.02	0.94–1.10	1.02	0.94–1.10
60–69			1.01	0.92–1.10	0.99	0.90–1.09	0.99	0.90–1.09
70+			1.05	0.95–1.17	1.06	0.95–1.19	1.06	0.95–1.19
Marital status legal (ref = Married)								
Divorced			1.20 ***	1.11–1.29	1.18 ***	1.10–1.28	1.18 ***	1.10–1.28
Never married			1.12 ***	1.05–1.18	1.13 ***	1.06–1.20	1.13 ***	1.06–1.20
Widowed			1.20 ***	1.12–1.29	1.19 ***	1.10–1.28	1.19 ***	1.10–1.28
Educational attainment level (ref = Tertiary)								
Primary			1.37 ***	1.27–1.47	1.29 ***	1.19–1.40	1.30 ***	1.20–1.40
Secondary			1.09 ***	1.04–1.14	1.08 ***	1.03–1.14	1.08 ***	1.03–1.14
Labour status (self-declared) (ref = Employed)								
Fulling domestic tasks			1.04	0.94–1.16	1.03	0.92–1.16	1.03	0.92–1.16
Other			1.03	0.94–1.12	1.11 *	1.00–1.22	1.11 *	1.00–1.22
Retired			1.52 ***	1.39–1.64	1.43 ***	1.31–1.56	1.44 ***	1.31–1.57
Unable to work due to long-standing health problem			2.41 ***	2.19–2.65	2.27 ***	2.05–2.52	2.27 ***	2.04–2.52
Unemployed			1.33 ***	1.22–1.46	1.33 ***	1.20–1.46	1.33 ***	1.20–1.46
Type of smoking behaviour (ref = No smoking)								
Daily smoking					0.94	0.89–1.00	0.95	0.89–1.00
Occasional smoking					1.04	0.94–1.16	1.04	0.94–1.16
Obesity status (ref = Normal weight)								
Obese					1.76 ***	1.66–1.86	1.76 ***	1.66–1.86
Pre-obese					1.13 ***	1.07–1.19	1.13 ***	1.07–1.19
Degree of urbanization (ref = Rural area/Thinly populated area)								
Cities or densely populated areas					1.13 ***	1.07–1.20	1.13 ***	1.07–1.19
Towns and suburbs/Moderately populated area					1.12 ***	1.06–1.19	1.12 ***	1.06–1.19
Carbon dioxide (ref = ‘≤0.900’)								
>0.925							0.66 **	0.49–0.88
]0.900–0.925]							0.78	0.54–1.15
PM_2.5_ [ref = ‘≤10’)								
>25							0.52	0.24–1.12
]10–25]							0.54 ***	0.38–0.78
Azote dioxide NO_2_ (ref = ‘≤10’)								
>20							1.18	0.67–2.07
]10–20]							0.92	0.62–1.37
Ozone O_3_ (ref = ‘≤2500’)								
>5000							1.43	0.67–2.07
]2500–5000]							0.92	0.62–1.37
Error Variance (Random Effects)								
Level-2 Intercept	0.1671 (1.1829)		0.1833 (1.2012)		0.1762 (1.1927)		0.0698 (1.0723)	
Model Fit								
-2LL	83,703		81,164		70,835		70,813	
AIC	83,707		81,200		70,883		70,877	

Note: (***) *p* < 0.001, (**) *p* < 0.01, and (*) *p* < 0.05.

**Table 4:** Estimates for the random effects (intercept) by country to have asthma in Europe, wave 3.

Subject	Estimate	Std. Error Predicted	95% Confidence Interval		*p*-Value
			Lower	Upper	
Austria	−0.0690	0.1351	−0.3338	0.1957	0.6093
Belgium	0.1084	0.1790	−0.2424	0.4592	0.5448
Bulgaria	−0.2511	0.1560	−0.5569	0.0547	0.1075
Cyprus	−0.1045	0.1402	−0.3793	0.1703	0.4561
Czech Republic	0.1897	0.1483	−0.1010	0.4804	0.2010
Denmark	0.3356 *	0.1508	0.0400	0.6313	0.0261
Estonia	−0.7579 ***	0.1778	−1.1063	−0.4095	<0.0001
Greece	0.0043	0.2642	−0.5135	0.5221	0.9869
Spain	−0.2777	0.1928	−0.6555	0.1002	0.1498
Croatia	−0.0466	0.1369	−0.3149	0.2218	0.7339
Hungary	0.1777	0.1354	−0.0877	0.4430	0.1895
Ireland	0.1208	0.1762	−0.2247	0.4662	0.4932
Iceland	0.2104	0.1554	−0.0941	0.5149	0.1756
Lithuania	−0.0795	0.1909	−0.4537	0.2947	0.6771
Luxembourg	−0.0580	0.1714	−0.3939	0.2779	0.7350
Latvia	−0.3696 *	0.1682	−0.6992	−0.0400	0.0280
Malta	0.2870 *	0.1376	0.0174	0.5567	0.0369
Netherlands	0.1700	0.1932	−0.2087	0.5486	0.3790
Norway	0.0756	0.1538	−0.2258	0.3769	0.6231
Poland	0.0550	0.1465	−0.2321	0.3421	0.7074
Portugal	0.1451	0.1824	−0.2125	0.5027	0.4264
Romania	−0.4593 **	0.1489	−0.7513	−0.1674	0.0020
Sweden	0.3042 *	0.1497	0.0107	0.5976	0.0422
Slovenia	0.0416	0.1371	−0.2272	0.3104	0.7616
Slovakia	0.2817	0.1546	−0.0212	0.5847	0.0684

Note: (***) *p* < 0.001, (**) *p* < 0.01, and (*) *p* < 0.05.

## Data Availability

The data supporting the findings of this study were provided by Eurostat and are available upon reasonable request, in accordance with Eurostat’s data access regulations.: https://ec.europa.eu/eurostat/web/microdata/european-health-interview-survey (accessed on 11 June 2024). All data generated or analysed during this study are included in this article.

## Appendix A

### Appendix A.1

**Table A1:** Sample size per country, Adults aged 20+.

	EHIS, Wave III (Realised in 2019)
Austria	14,606
Belgium	9119
Bulgaria	7198
Cyprus	5762
Czech Republic	7656
Denmark	6279
Estonia	4635
Greece	7845
Spain	21,238
Croatia	5252
Hungary	5302
Ireland	7196
Iceland	3601
Lithuania	4656
Luxembourg	4280
Latvia	5550
Malta	4205
Netherlands	7620
Norway	7442
Poland	18,957
Portugal	14,112
Romania	15,280
Serbia	12,426
Sweden	9075
Slovenia	9075
Slovakia	5356

Source: Eurostat, Wave III of EHIS, Conducted in 2019.

### Appendix A.2

**Table A2:** Data on climate and environmental risks used in this study.

Wave 3	Carbon Dioxide (CO_2_)	Particulate Matter (PM_2.5_)	Azote Dioxide (NO_2_)	Ozone (O_3_)
Austria	0.902	12.3	18.9	5311
Belgium	0.895	12.5	20.9	2553
Bulgaria	0.922	22.4	19.2	3938
Cyprus	0.922	15.7	19.6	6029
Czech Republic	0.880	17.1	15.2	4307
Denmark	0.934	8.5	8.8	1711
Estonia	0.885	5.4	6.3	1462
Greece	0.927	30.0	23.6	4858
Spain	0.935	12.0	21.6	5600
Croatia	0.940	17.6	15.6	7110
Hungary	0.927	18.8	17.8	5010
Ireland	0.902	6.2	9.3	1418
Iceland	0.875	5.1	10.2	782
Lithuania	0.926	10.3	10.8	1417
Luxembourg	0.810	10.0	19.5	3001
Latvia	0.948	9.5	11.1	1557
Malta	0.947	11.8	16.0	6174
Netherlands	0.883	11.3	20.2	2281
Norway	0.889	5.2	10.4	1448
Poland	0.885	21.4	14.9	3111
Portugal	0.942	9.1	16.2	3914
Romania	0.946	17.9	18.8	3885
Serbia	0.928	28.2	20.6	4293
Sweden	0.944	5.0	7.7	1641
Slovenia	0.912	16.2	16.2	7035
Slovakia	0.918	18.8	14.7	4861

### Appendix A.3

**Table A3:** Prevalence of asthma by sex and educational level.

Wave 3	Men					Women				
	%	Primary	Secondary	Tertiary	*p*-Value	%	Primary	Secondary	Tertiary	*p*-Value
Austria	4.2	8.3	4.4	3.7	0.1318	4.5	12.2	4.8	3.4	<0.0001
Belgium	5.0	8.7	5.4	3.7	0.0003	6.2	7.1	6.5	5.4	0.2090
Bulgaria	1.9	6.5	1.6	2.1	0.0002	2.7	4.8	2.7	2.1	0.0431
Cyprus	3.2	5.6	2.9	2.8	0.0276	5.0	7.6	5.2	3.5	0.0020
Czech Republic	3.3	6.2	3.5	2.4	0.3313	6.1	12.8	6.9	3.4	0.0007
Denmark	6.7	4.8	7.7	5.8	0.0901	7.9	9.3	10.0	6.1	0.0007
Estonia	3.4	4.7	3.9	2.4	0.2275	4.5	6.9	5.1	3.6	0.1382
Greece	2.4	4.0	2.2	1.6	0.0038	4.4	6.6	4.2	2.7	<0.0001
Spain	3.4	3.9	3.1	3.6	0.1499	4.7	5.5	4.7	3.9	0.0105
Croatia	4.0	4.3	3.8	4.8	0.6074	5.4	9.4	5.2	4.2	0.0024
Hungary	4.0	4.0	4.1	3.8	0.9437	6.0	19.4	6.0	4.8	<0.0001
Ireland	7.3	7.6	7.8	6.9	0.6273	7.1	9.7	6.7	7.0	0.1258
Iceland	6.7	8.0	7.4	4.6	0.0701	13.4	14.5	12.5	13.3	0.6008
Lithuania	1.6	3.2	1.2	2.2	0.1554	3.8	6.2	4.1	3.0	0.1412
Luxembourg	5.1	2.8	4.8	5.3	0.5372	6.7	12.7	5.9	6.9	0.0262
Latvia	3.2	2.5	3.3	2.9	0.8446	4.3	7.9	4.8	3.0	0.0145
Malta	5.1	4.9	5.4	5.3	0.8684	7.0	6.6	8.3	6.9	0.5809
Netherlands	5.8	8.0	6.2	4.3	0.0126	7.0	12.6	7.1	5.4	<0.0001
Norway	6.3	7.0	6.8	5.5	0.3022	9.2	0.0	10.2	8.7	0.1755
Poland	3.3	6.8	2.8	3.2	<0.0001	4.9	7.8	5.0	3.3	<0.0001
Portugal	4.7	5.7	3.9	4.3	0.0082	6.9	8.9	5.4	5.7	<0.0001
Romania	1.2	2.5	1.4	0.2	0.0003	1.9	5.2	1.6	1.2	<0.0001
Serbia	3.5	8.3	3.5	2.5	<0.0001	3.9	8.6	3.6	2.3	<0.0001
Sweden	5.9	5.3	6.3	5.5	0.5319	8.9	11.7	9.2	8.2	0.1230
Slovenia	4.3	10.5	5.4	3.7	0.0034	5.2	7.5	5.7	4.8	0.3246
Slovakia	2.7	34.9	2.6	2.6	<0.0001	5.5	6.4	6.0	4.1	0.1778

In wave 3, the prevalence of asthma ranged from 1.9% (in Romania) to 13.4% (in Iceland) among women and from 1.2% (in Romania) to 7.3% (in Ireland) among men. A statistically significant negative gradient across educational levels was observed among men in countries such as Belgium, Bulgaria, Cyprus, Greece, the Netherlands, Poland, Portugal, Romania, Serbia, Slovenia, and Slovakia. That is, the higher the level of education attained, the lower the prevalence of asthma. Among women, this negative gradient was observed in Austria, Bulgaria, Cyprus, Czech Republic, Denmark, Greece, Spain, Croatia, Hungary, Luxembourg, Latvia, the Netherlands, Poland, Portugal, Romania, and Serbia.

### Appendix A.4

**Table A4:** Estimates of socioeconomic and environmental determinants of asthma prevalence in European countries (wave 3, 2019), based on unweighted data.

Variables	Model 0		Model 1		Model 2		Model 3	
	OR	95%CI	OR	95%CI	OR	95%CI	OR	95%CI
Fixed Effects								
Intercept	0.05 ***	0.04–0.06	0.03 ***	0.02–0.04	0.03 ***	0.02–0.03	0.04 ***	0.03–0.06
Sex (ref = Male)								
Female			1.33 ***	1.28–1.38	1.37 ***	1.32–1.42	1.37 ***	1.32–1.42
Age-group (ref = 30–39)								
20–29			0.99	0.91–1.08	1.03	0.95–1.12	1.03	0.95–1.12
40–49			1.04	0.97–1.11	1.03	0.96–1.10	1.03	0.96–1.10
50–59			1.06	0.99–1.13	1.01	0.94–1.09	1.01	0.94–1.09
60–69			1.04	0.97–1.13	0.99	0.91–1.08	0.99	0.92–1.07
70+			1.09 *	1.00–1.19	1.06	0.97–1.16	1.06	0.97–1.16
Marital status legal (ref = Married)								
Divorced			1.16 ***	1.09–1.23	1.14 ***	1.07–1.22	1.14 ***	1.07–1.22
Never married			1.05	0.99–1.10	1.06 *	1.01–1.12	1.06 *	1.01–1.12
Widowed			1.14 ***	1.08–1.21	1.15 ***	1.08–1.22	1.15 ***	1.08–1.22
Educational attainment level (ref = Tertiary)								
Primary			1.45 ***	1.36–1.54	1.40 ***	1.30–1.49	1.40 ***	1.30–1.50
Secondary			1.12 ***	1.07–1.16	1.09 ***	1.05–1.14	1.09 ***	1.05–1.14
Labour status (self-declared) (ref = Employed)								
Fulling domestic tasks			1.04	0.96–1.13	1.03	0.95–1.13	1.04	0.95–1.13
Other			1.11 **	1.03–1.21	1.18 ***	1.08–1.29	1.18 *	1.08–1.29
Retired			1.33 ***	1.24–1.42	1.30 ***	1.21–1.39	1.30 ***	1.21–1.39
Unable to work due to long-standing health problem			2.37 ***	2.17–2.59	2.30 ***	2.10–2.53	2.30 ***	2.09–2.53
Unemployed			1.23 ***	1.13–1.33	1.23 ***	1.13–1.34	1.23 ***	1.13–1.34
Type of smoking behaviour (ref = No smoking)								
Daily smoking					0.95	0.90–1.00	0.95	0.90–1.00
Occasional smoking					1.08	0.98–1.18	1.08	0.98–1.18
Obesity status (ref = Normal weight)								
Obese					1.77 ***	1.68–1.85	1.77 ***	1.68–1.85
Pre-obese					1.16 ***	1.11–1.21	1.16 ***	1.11–1.21
Degree of urbanization (ref = Rural area/Thinly populated area)								
Cities or densely populated areas					1.15 ***	1.10–1.20	1.15 ***	1.09–1.20
Towns and suburbs/Moderately populated area					1.09 ***	1.04–1.14	1.09 ***	1.04–1.14
Carbon dioxide (ref = ‘≤0.900’)								
>0.925							0.63 **	0.47–0.83
]0.900–0.925]							0.70 *	0.50–0.99
PM_2.5_ (ref = ‘≤10’)								
>25							0.69	0.32–1.44
]10–25]							0.65 *	0.46–0.92
Azote dioxide NO_2_ (ref = ‘≤10’)								
>20							0.97	0.57–1.63
]10–20]							0.89	0.60–1.33
Ozone O_3_ (ref = ‘≤2500’)								
>5000							1.37	0.92–2.03
]2500–5000]							0.96	0.69–1.34
Error Variance (Random Effects)								
Level-2 Intercept	0.1291 *** (1.1378)		0.1429 (1.1536)		0.1388 *** (1.1489)		0.0703 *** (1.0728)	
Model Fit								
-2LL	115,068		112,255		100,626		100,608	
AIC	115,072		112,291		100,674		100,672	

Note: (***) *p* < 0.001, (**) *p* < 0.01, and (*) *p* < 0.05.
